# Supplementary material for: 53BP1 can limit sister-chromatid rupture and rearrangements driven by a distinct ultrafine DNA bridging-breakage process
Source: Nat Commun. 2018 Feb 14;9:677. doi: 10.1038/s41467-018-03098-y (PMC5813243; doi:10.1038/s41467-018-03098-y)
Supplement: Supplementary file 1 — Supplementary Information [file 41467_2018_3098_MOESM1_ESM.pdf]

Supplementary Information

53BP1 can limit sister-chromatid rupture and rearrangements driven by a distinct ultrafine DNA bridging-breakage process

Tiwari et al.

Supplementary Figure 1

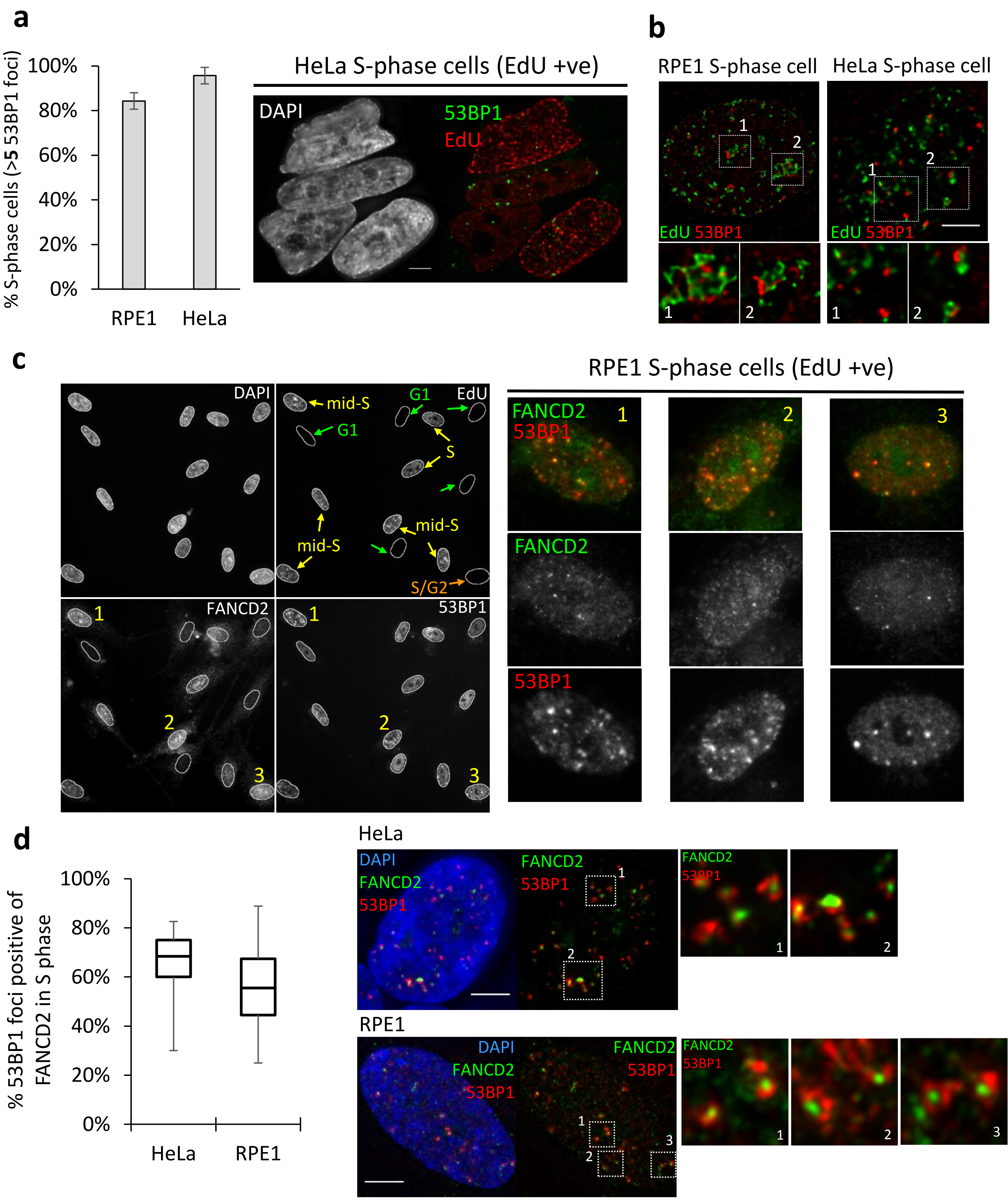

Supplementary Figure 1. Adjacent co-localisation of FANCD2 and 53BP1 during S phases of normal and cancer cells. (a) Quantification of 53BP1 foci formation in unperturbed RPE1 and HeLa S-phase cells **(Left)**. EdU is used to label S-phase cells. Numbers of S-phase cells counted: RPE1=794, HeLa=611 from 3 independent experiments. 53BP1 foci (green) are detected in EdU-labelled (red) HeLa cells **(Right)**. Error bars represent s.d.

(b) 53BP1 (red) localised adjacently to newly synthesised DNA regions pulse-labelled by EdU (green), in RPE1 **(Left)** and HeLa **(Right)** cells. Inset shows enlarged view of the indicated regions.

(c) 53BP1 localised adjacently to FANCD2 foci in S phase cells. RPE1 cells were EdU labelled and immunostained for FANCD2 and 53BP1. Representative images showing cells in different cell cycle phases (green arrows: G1; yellow: S; orange: S/G2). Nuclei were outlined based on DAPI staining. Right panels showing enlarged views of the indicated cells and the co-localisation of 53BP1 (red) and FANCD2 (green) foci in S phase.
(d) Left: A box plot showing the percentage of 53BP1 foci positive of FANCD2 in S-phase HeLa and RPE1 S-phase cells. 20 to 30 z-stacking images were acquired in unperturbed S-phase cells. Total numbers of 53BP1 foci in HeLa (n=796 foci) and in RPE1 (n=601 foci) are scored in 30 S-phase cells. Error bars represent s.e.m. **Right:** Representative examples of 53BP1 proteins (red) accumulating around FANCD2 (green) foci in unperturbed HeLa and RPE1 cells. Insets show enlarged views of the indicated regions.

Supplementary Figure 2

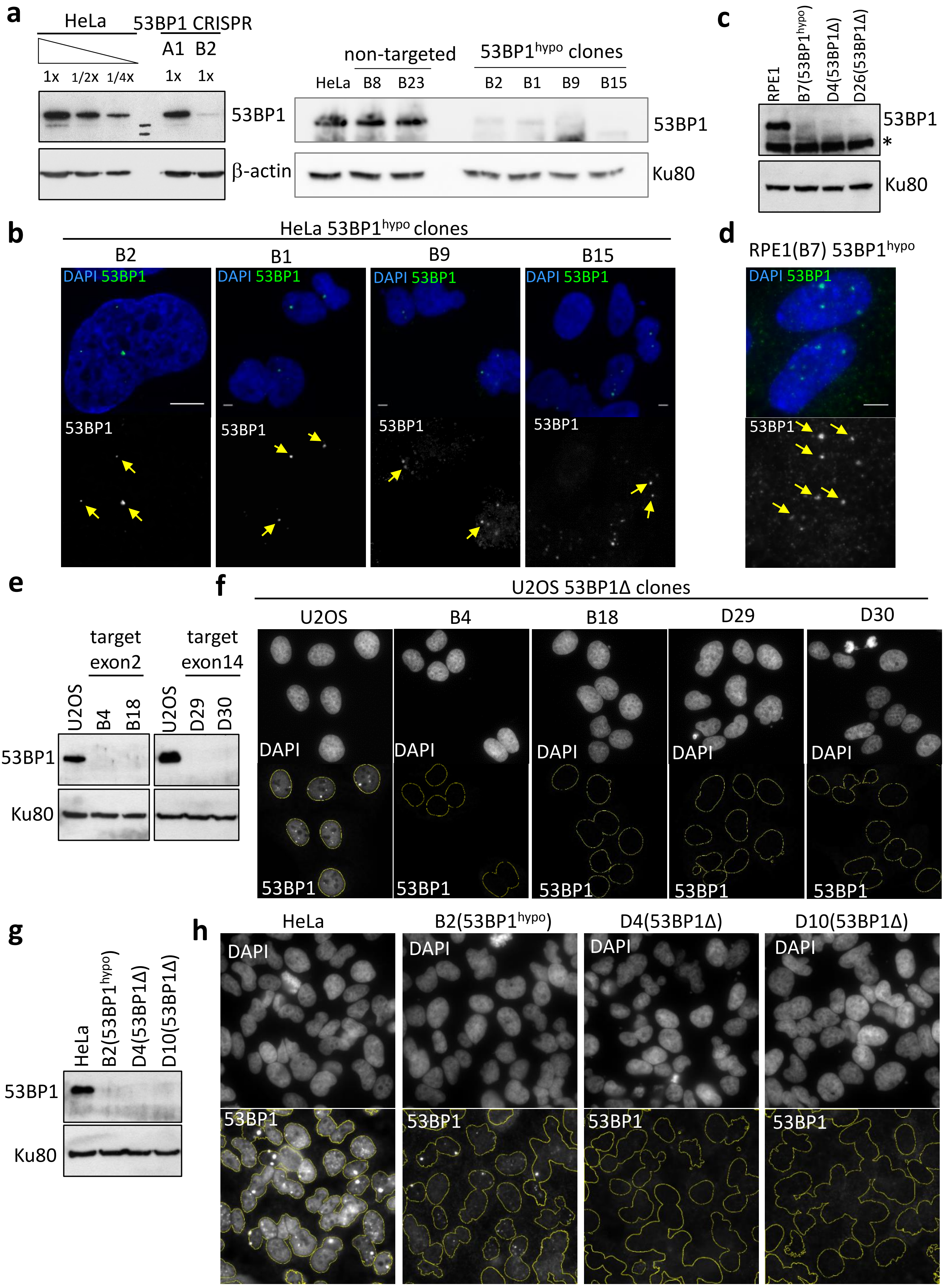

Supplementary Figure 2. Verification of 53BP1^{hypo} and 53BP1Δ clones in HeLa, RPE1 and U2OS cells by immunoblotting and immunofluorescence staining.

(a) Residual expression of a full-length like 53BP1 protein in CRISPR/CAS9 knockout clones. **Left**: B2 (53BP1^{hypo}) clone showing ~15% of 53BP1 levels as compared to HeLa. β-actin was used as a loading control. **Right**: Immunoblotting of 53BP1 protein levels in HeLa, 53BP1 non-targeted (b8, b23) and targeted clones (B2, b1, b9, b15). Ku80 was used as a loading control.

(b) Representative immunofluorescent images of HeLa 53BP1^{hypo} clones (B2, b1, b9 and b15) showing small 53BP1 nuclear focal formation (arrows).

(c) Immunoblotting of 53BP1 proteins in 53BP1^{hypo} and 53BP1Δ clones in RPE1 cells. Ku-80 was used as a loading control. Asterisk indicates a non-specific band.

(d) Representative immunofluorescent image of RPE1 B7 (53BP1^{hypo}) clone showing small 53BP1 foci (arrows).

(e) Immunoblotting of 53BP1 protein in CRISPR/CAS9 clones of U2OS by targeting exon 2 (left) and exon 14 (right). Ku-80 was used as a loading control.

(f) Representative IF images of parental U2OS and U2OS 53BP1Δ clones (B4, B18, D29 and D30). Nuclei are outlined (yellow).

(g) Immunoblotting of 53BP1 protein in HeLa B2 (53BP1^{hypo}) and D4 and D10 (53BP1Δ) cells. Ku-80 was used as a loading control.

(h) Representative IF images of HeLa 53BP1^{hypo} (B2) and 53BP1Δ (D4 and D10) clones. Note the formation of weak and small 53BP1 nuclear foci in 53BP1^{hypo} cells, but not in 53BP1Δ cells. Nuclei are outlined (yellow).

Supplementary Figure 3

a

53BP1 (EXON 2)

gRNA PAM

WT (0%) GCCTGAAAGCCAGGTTCTAGAGGATGATTCTGGTTCTCACTTCAGTATGCT

Type I (47%) GCCTGAAAGCCA - GGTTCTCACTTCAGTATGCT

Type II (23%) GCCTGAA- -TTCTGGTTCTCACTTCAGTATGCT

Type III (30 %) GCCTGAAAGCCAGGTTCTAGAGGATGTGATTCTGGTTCTCACTTCAGTATGCT

b

WT-allele specific primer

WT sequence GCCTGAAAGCCAGGTTCTAGAGGATGATTCTGGTTCTCACTTCAGTATGCT

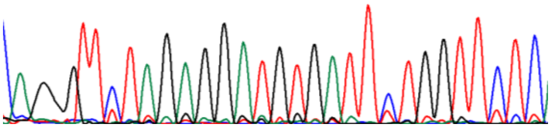

Type III mutation CAGGTTCTAGAGGATGTGATTCTGGTTCTC

c

Type I mutant product:
MPGEQMDPTGSQLDSDFSQQDTPCLIIEDSQPESQVLTSVCYLDTFLISRRTKKILCWMLCPILNKQ
LEKNEETVIVGSMNI*

Type II mutant product:
MPGEQMDPTGSQLDSDFSQQDTPCLIIEDSQPEFWFSLQYAISTPS*

Type III mutant product:
MPGEQMDPTGSQLDSDFSQQDTPCLIIEDSQPESQVLEDVILVLTSVCYLDTFLISRRTKKILCWML
CPILNKQLEKNEETVIVGSMNI*

d

Isoform 3 Isoform 1 CRISPR mutation sites Alternative start site

MPGEQMDPTGSQLDSDFSQQDTPCLIIEDSQPESQVLEDDSGSHFSMLSRHLPNLQTHKENPVLDVVSNP
EQTAGEERGDGNSGFNEHLKENKVADPVDSSNLDTCGSISQVIEQLPQPNRTSSVLGMSVESAPAVEEEKGE
ELEQKEKEKEEDTSGNTTHSLGAEDTASSQLGFGVLELSQSQDVEENTVPYEVDKEQLQSVTTNSGYTRLSD
VDANTAIKHEEQSNEDIPIAEQSSKDIPVTAQPSKDVHVVKEQNPPPARSEDMPFSPKASVAAMEAKEQLSAQ
ELMESGLQIQKSPEPEVLSTQEDLFDQSNKTVSSDGCSTPSREEGGCSLASTPATTLHLLQLSGQRSLVQDSL
STNSSDLVAPSPDAFRSTPFIVPSSPTEQEGRQDKPMDTSVLSEEGGEPFQKKLQSGEPVELENPPLLPESTV
SPQASTPISQSTPVFPPGSLPIPSQPQFSHDIFIPSPSLEEQSNDGKKDGDMHSSSLTVECSKTSEIEPKNSPE
DLGLSLTGDSCKLMLSTSEYSQSPKMESLSSHRIDEDGENTQIEDTEPMSPVLNSKFVPAENDSILMNPAQDG
EVQLSQNDDKTKGDDTDTRDDISILATGCKGREETVAEDVCIDLTCDSGSQAVPSPATRSEALSSVLDQEEAM
EIKEHHPEEGSSGSEVEEIPETPCESQGEELKEENMESVPLHLSLTETQSQGLCLQKEMPKKECSEAMEVET
SVISIDSPQKLAILDQELEHKEQEAWEEATSEDSSVVIVDVKEPSPRVDVSCEPLEGVEKCSDSQSWEDIAPEI
EPCAENRLDTKEEKSVEYEGDLKSGTAETEPVEQDSSQPSLPLVRADDPLRLDQELQQPQTQEKTSNSLTED
SKMANAKQLSSDAEAQKLGKPSAHASQSFCESSSETPFHFTLPKEGDIIPPLTGATPPLIGHLKLEPKRHSTPI
GISNYPESTIATSDVMSESMVETHDPILGSGKGDSGAAPDVDDKLCLRMKLVSPETEASEESLQFNLEKPATG
ERKNGSTAVAESVASPQKTMSVLSCICEARQENEARSEDPPTTPIRGNLLHFPSSQGEEEKEKLEGDHTIRQS
QQPMKPISPVKDPVSPASQKMVIQGPSSPQGEAMVTDVLEDQKEGRSTNKENPSKALIERPSQNNIGIQTME
CSLRVPETVSAATQTIKNVCEQGTSTVDQNFGKQDATVQTERGSGEKPVSAPGDDTESLHSQGEEEFDMPQ
PPHGHVLHRHMRTIREVRTLVTRVITDVYYVDGTEVERKVTEETEEPIVECQECETEVSPSQTGGSSGDLGDI
SSFSSKASSLHRTSSGTSLSAMHSSGSSGKGAGPLRGKTSGTEPADFALPSSRGGPGKLSPRKGVSQTGTP
VCEEDGDAGLGIRQGGKAPVTPRGRGRRGRPPSRTTGTRETAVPGPLGIEDISPNLSPDDKSFSRVVPRVPD
STRRTDVGAGALRRSDSPEIPFQAAAGPSDGLDASSPGNSFVGLRVVAKWSSNGYFYSGKITRDVGAGKYKL
LFDDGYECDVLGKDILLCDPIPLDTEVTALSEDEYFSAGVVKGHRKESGELYYSIEKEGQRKWYKRMAVILSLE
QGNRLREQYGLGPYEAVTPLTKAADISLDNLVEGKRKRRSNVSSPATPTASSSSSTTPTRKITESPRASMGVL
SGKRKLITSEEERSPAKRGRKSATVKPVGAGEFVSPCESGDNTGEPSALEEQRGPLPLNKTLFLGYAFLLTMA
TTSDKLASRSKLPDGPTGSSEEEEEFLEIPPFNKQYTESQLRAGAGYILEDFNEAQCNTAYQCLLIADQHCRTR
KYFLCLASGIPCVSHVWVHDSCHANQLQNYRNYLLPAGYSLEEQRILDWQPRENPFQNLKVLLVSDQQQNFL
ELWSEILMTGGAASVKQHHSSAHNKDIALGVFDVVVTDPSCPASVLKCAEALQLPVVSQEWVIQCLIVGERIGF
KQHPKYKHDYVSH*

Santa Cruz 53BP1 antibody against 1-300 amino acids

Abcam 53BP1 antibody against 390-900 amino acids

Supplementary Figure 3. DNA sequence analyses of 53BP1 hypomorphic allele.

(a) Sequence of the sgRNA highlighted in red used to generate 53BP1 hypomorphic cells ($53BP1^{hypo}$). Arrow indicates the CRISPR/CAS9 cleavage site. Sanger sequencing result, following TA-cloning, of 40 individual plasmids identified three different types of mutations in 53BP1 exon 2 in HeLa B2 clone.

(b) No wildtype (WT) 53BP1 exon 2 sequence was recovered after WT allele-specific PCR amplification. A primer annealing only to wildtype exon 2 sequence was designed to PCR amplify any remaining WT allele after CRISPR/CAS9 gene editing. Chromatogram of DNA sequence obtained by wildtype (WT) allele-specific PCR amplification showing only type III mutation (2 bases insertion) without any mixed reads of WT sequences.

(c) Translations of the mutant alleles generating premature termination peptides. All the mutations lead to a premature STOP codon.

(d) A potential hypomorphic 53BP1 isoform translated from methionine 47. Known 53BP1 isoforms 1 and 3 from UniProt database were depicted. Antibodies against the first 300 residues, plus from residues 390-900 of 53BP1 were shown on the right.

Supplementary Figure 4

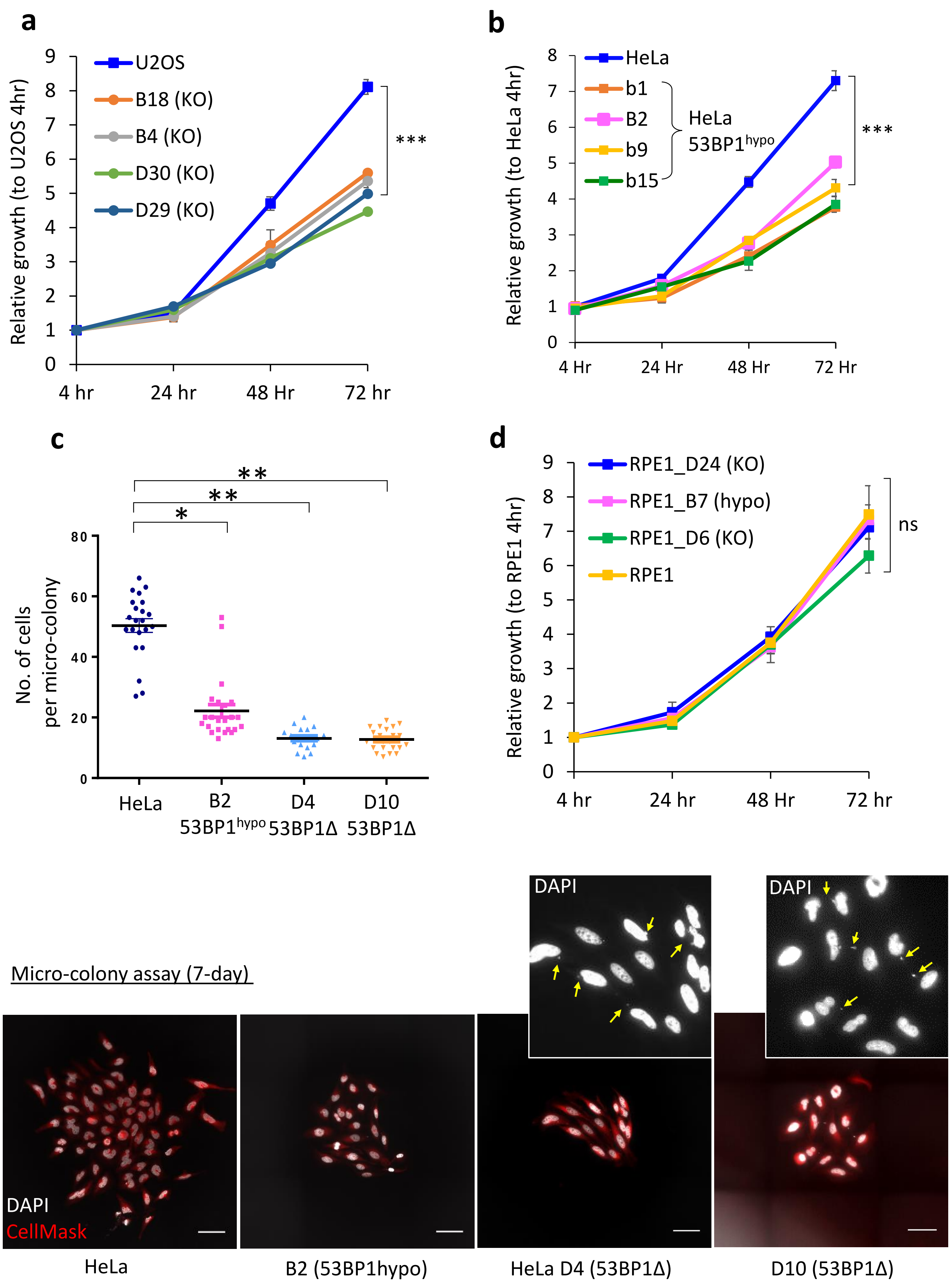

Supplementary Figure 4. 53BP1Δ and 53BP1^{hypo} cancer cells show increased proliferation defects.
(a) Growth curves of U2OS and U2OS 53BP1Δ clones (B18, B4, D30, D29) determined by MTT assays. Error bars represent s.d of three independent experiments. Statistical significance was determined by two-way ANOVA (***p<0.005).
(b) Growth curves of HeLa and HeLa 53BP1^{hypo} clones (b1, B2, b9 and b15) determined by MTT assays. Other details as **(a).**
(c) Micro-colony growth assay showing severe proliferation defects in HeLa 53BP1Δ cells. HeLa, B2 (53BP1^{hypo}), D4 and D10 (53BP1Δ) cells were seeded onto cover glass in limited dilution and allowed to form micro-colonies. Number of cells in each single, well-separated colony was counted after 7 days. Cells in over 20 micro-colonies in each cell line were counted (**top**). Images showing micro-colonies of HeLa, B2 (53BP1^{hypo}), D4 and D10 (53BP1Δ) cells stained with DAPI (grey) and cell mask dye (red) (**bottom**). Note, more severe cell growth defects present in HeLa 53BP1Δ clones. Statistical significance was determined by T-test (*, $p < 0.05$; **, $p < 0.01$).
(d) Growth curves of RPE1, B7 (53BP1^{hypo}), D6 and D24 (53BP1Δ) clones determined by MTT assays. Other details as **(a)** (ns: non-significant).

Supplementary Figure 5

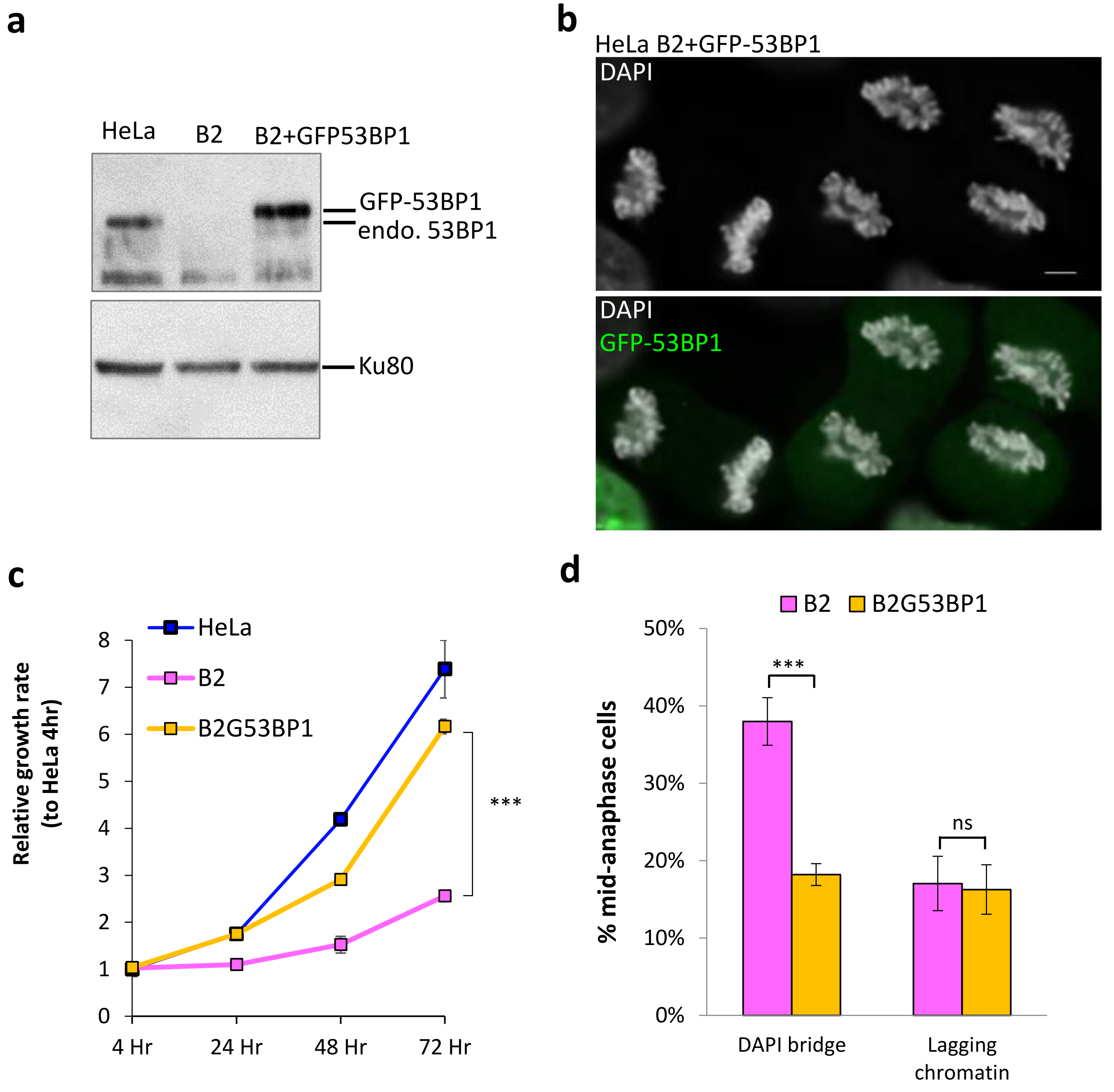

Supplementary Figure 5. Ectopic overexpression of EGFP-tagged 53BP1 in HeLa 53BP1^{hypo} cells rescues slow growth and chromosome missegregation phenotypes.

(a) Generation of stable HeLa B2 (53BP1^{hypo}) cells ectopically overexpressing an EGFP-tagged full-length 53BP1. Immunoblotting showing marginal overexpression of the EGFP-53BP1 in HeLa B2 (53BP1^{hypo}) cells (B2G53BP1). Ku80 was used as a loading control.

(b) Representative images of HeLa B2G53BP1 anaphase cells in the same field showing normal complete chromosome disjunction. Scale bars, 5 µm.

(c) Ectopic expression of EGFP-53BP1 partially rescued the slow growth phenotype in B2(53BP1^{hypo}) cells. Growth curves of HeLa, B2 (53BP1^{hypo}) and B2G53BP1 (B2+EGFP-53BP1) determined by MTT assays. Error bars represent s.d. of three independent experiments. Statistical significance was determined by two-way ANOVA (***$p<0.005$).

(d) Ectopic expression of EGFP-53BP1 reduced anaphase bridge formation in HeLa B2 (53BP1^{hypo}) cells. A graph showing the percentages of anaphase cells with chromatin bridges and lagging chromatin. Total numbers of anaphase cells counted: B2=352, B2G53BP1=310 from 3 separate preparations. Error bars represent s.d. Note: Percentages of B2 and B2G53BP1 cells having lagging chromatin are similar, see Fig. 5g-h for explanation). Statistical significance was determined by T-test (***, *p* < 0.001; *ns*, nonsignificant).

Supplementary Figure 6

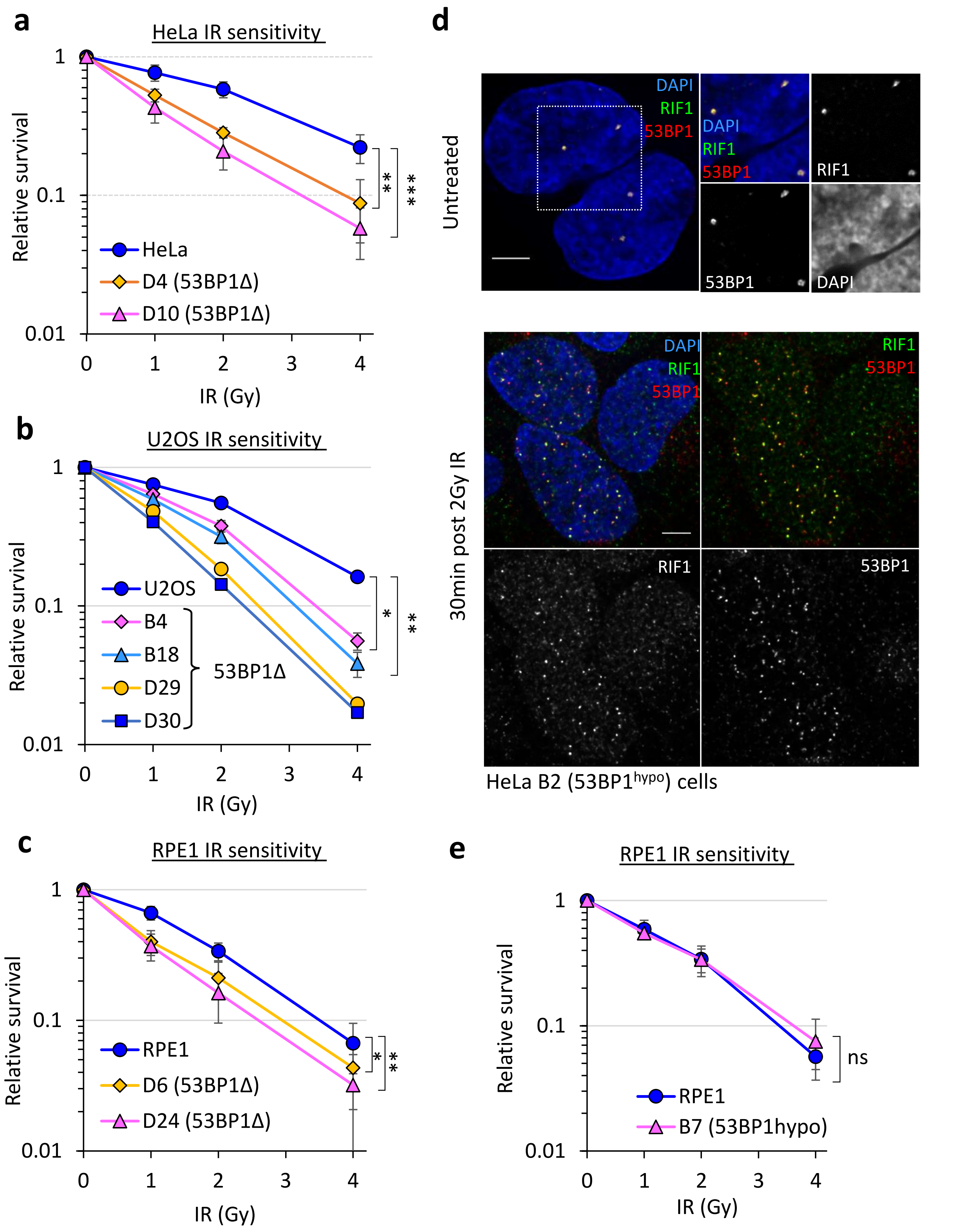

Supplementary Figure 6. Increased IR sensitivities in 53BP1 complete knockout, but not 53BP1^{hypo}, cells. Relative survival curves after indicated doses of IR treatments in HeLa 53BP1Δ **(a)**, U2OS 53BP1Δ **(b)**, and RPE1 53BP1Δ **(c)** clones. **(d)** Co-localisation of RIF1 (green) and 53BP1 (red) in untreated (top) and IR-treated (bottom) HeLa B2 (53BP1^{hypo}) cells. **(e)** Relative survival curves after indicated doses of IR treatments in RPE1 53BP1^{hypo} cells. Error bars represent s.d. from three independent experiments. Statistical significance was determined by two-way ANOVA. (*p<0.05, **p<0.01, ***p<0.005, *ns*: non-significant).

Supplementary Figure 7

a

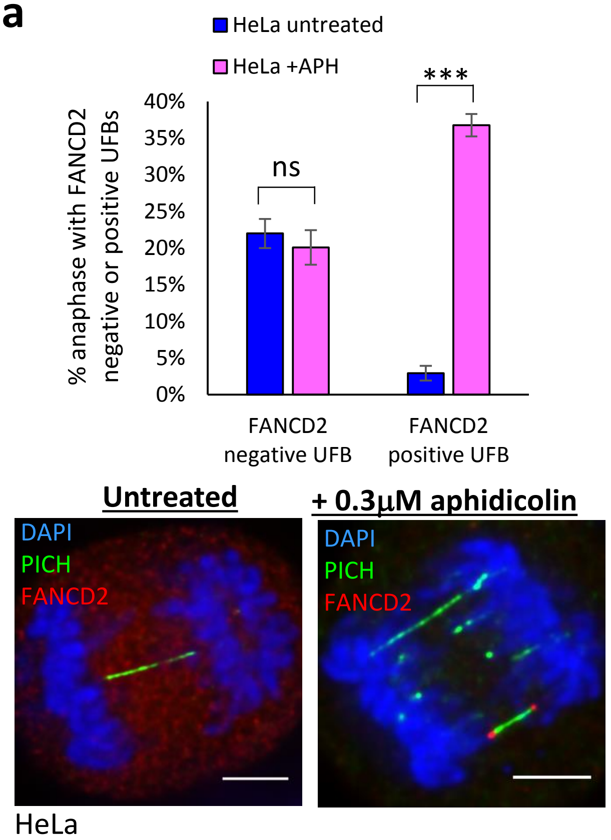

b

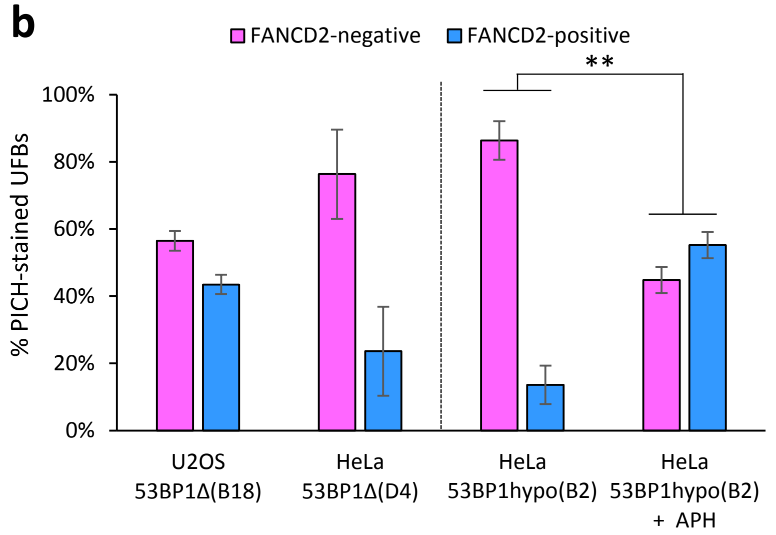

c

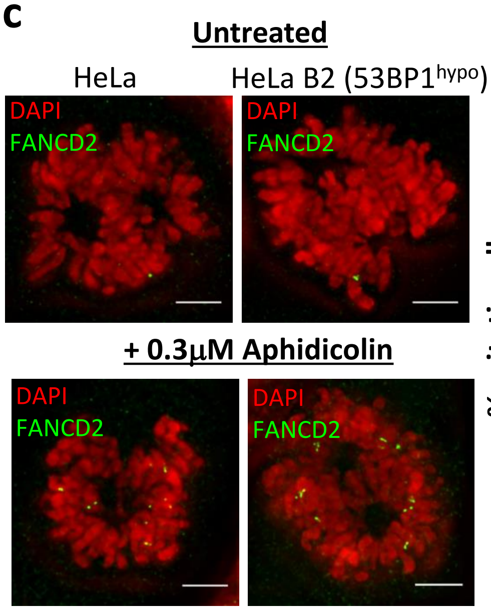

d

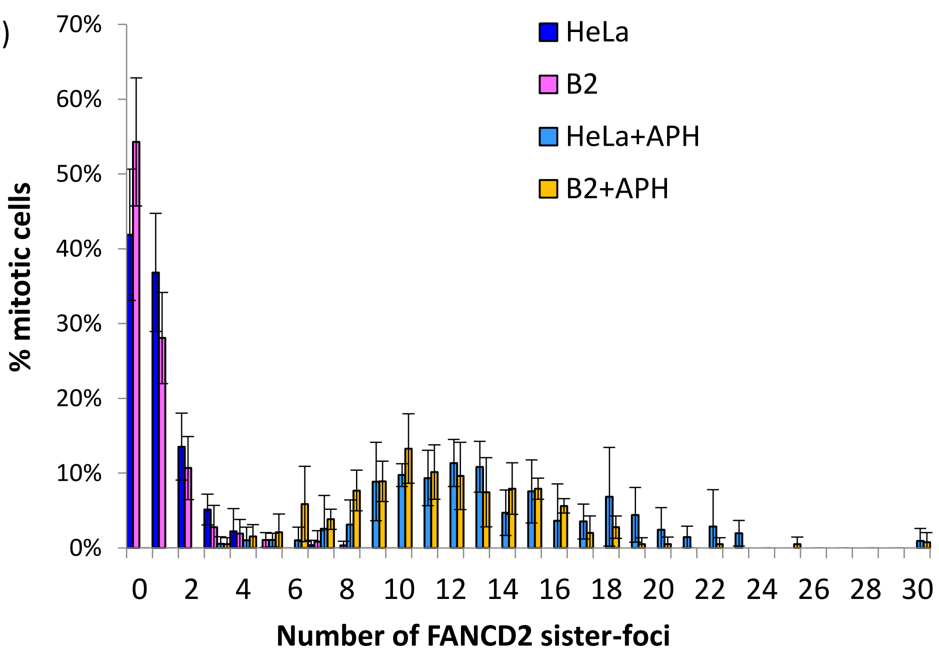

e

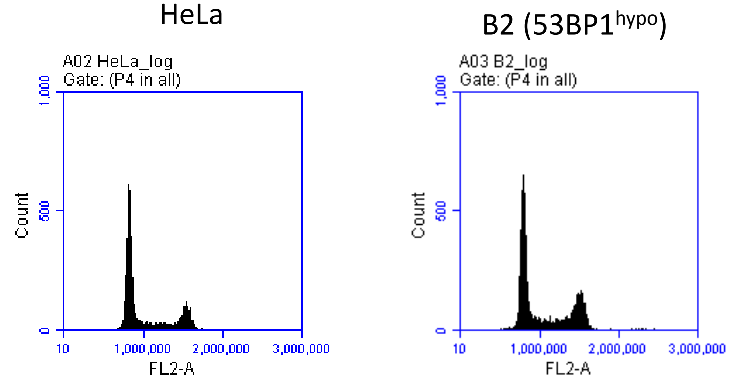

f

FRA16D CFS expression

No. of FRA16D with break	HeLa	B2 (53BP1^{hypo})
	0/87	0/76

Supplementary Figure 7. Fanconi anemia pathway is proficient in 53BP1-depleted cells in response to aphidicolin-induced replication stress

HeLa B2 ($53BP1^{hypo}$) cells did not show increased levels of spontaneous FANCD2 mitotic foci but responded to low dose aphidicolin treatment for the formation of FANCD2 mitotic foci.

(a) A low dose of aphidicolin treatment [0.3μM] significantly increased the frequency of FANCD2 positive, but not FANCD2 negative, UFBs in HeLa anaphase cells. Numbers of anaphase cells counted: HeLa untreated=135, HeLa+APH=120 from 3 independent experiments. Representative images are shown below. Scale bars, 5 μm. Statistical significance was determined by T-test (***p<0.001).

(b) Quantitation of PICH-UFBs positive and negative for FANCD2 in U2OS 53BP1Δ (B18), HeLa 53BP1Δ (D4) and HeLa $53BP1^{hypo}$ (B2). Mid-anaphase cells were examined either treated or not treated with 0.3uM aphidicolin for 16hr. Note, a majority of PICH-UFB stained negative for FANCD2 in 53BP1 depleted cells. Over 100 PICH-UFBs were scored from 3 independent experiments. Statistical significance was determined by T-test (**p<0.01).

(c) Z-projection high-resolution images of FANCD2 focal formation in HeLa and B2 ($53BP1^{hypo}$) pro-metaphase cells, with and without aphidicolin treatments. Scale bars, 5 μm.

(d) Frequencies of mitotic cells with FANCD2 sister foci in untreated, or aphidicolin-treated conditions, in both HeLa and B2 ($53BP1^{hypo}$) populations. Total numbers of mitotic cells counted: HeLa=227, B2=215, HeLa+APH=179, B2+APH=174. Error bars represent s.d of three independent experiments.

(e) Cell cycle profiles of exponentially growing HeLa and B2 ($53BP1^{hypo}$) cells.

(f) A table illustrating FRA16D common fragile site (CFS) expression in HeLa and B2 ($53BP1^{hypo}$) chromosomes. FISH was performed by using BAC-264L1 probe hybridising FRA16D site.

Supplementary Figure 8

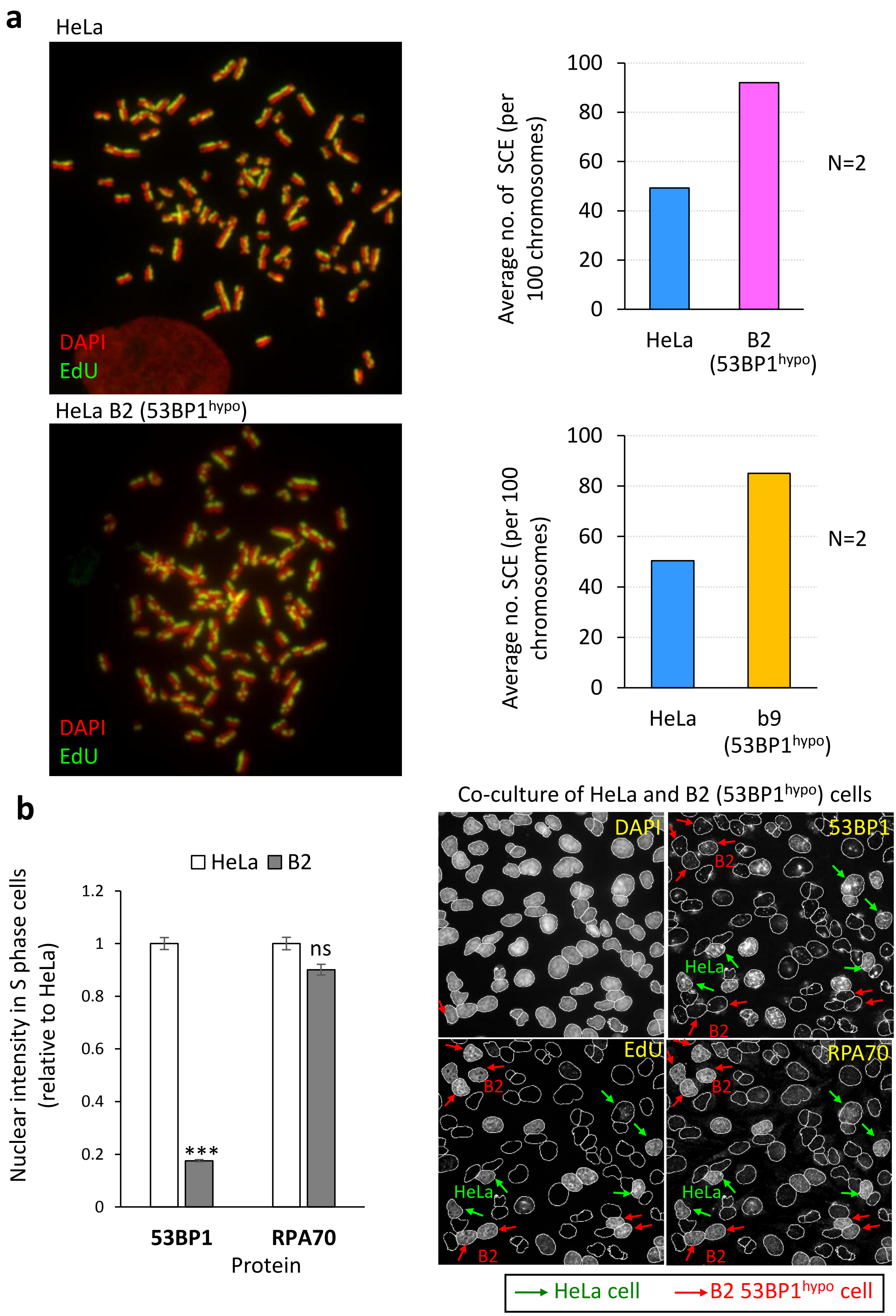

Supplementary Figure 8. 53BP1 hypomorphic cells display increased rates of sister chromatid exchange (SCE). (a) Sister chromatids were differentially labelled by using EdU (green) in HeLa, B2 and b9 ($53BP1^{hypo}$) cells. Graphs showing quantitation of average SCE rates in HeLa, B2 and b9 ($53BP1^{hypo}$) cells (**right**). Over 2000 chromosomes were scored from 2 independent experiments. Representative metaphase spreads showing SCEs in HeLa and B2 $53BP1^{hypo}$ cells are shown (**left**).

(b) Nuclear intensities of 53BP1 and RPA70 protein in HeLa and B2 ($53BP1^{hypo}$) S-phase cells (left). HeLa and B2 ($53BP1^{hypo}$) cells were mixed and seeded together on the same coverglasses. Co-cultured cells were pulsed labelled with EdU for 15min and then pre-extracted before subjecting to IF staining. Using ScanR imaging analysis, B2 ($53BP1^{hypo}$) population (red arrow) were separated from HeLa (green arrow) based on 53BP1 expression intensities (a 5-fold difference). S-phase cells were selected based on the presence of EdU signal. RPA intensities were compared in the sorted S-phase populations of HeLa and B2 ($53BP1^{hypo}$) cells. Right: representative images of the co-cultured HeLa and B2 ($53BP1^{hypo}$) cells stained with EdU, 53BP1 and RPA. Nuclei were outlined based on DAPI staining. HeLa (n=288) and B2 (n=438) EdU +ve cells were measured. Error bars represent s.e.m. Statistical significance was determined by T-test (***$p < 0.001$; ns, nonsignificant).

Supplementary Figure 9

a

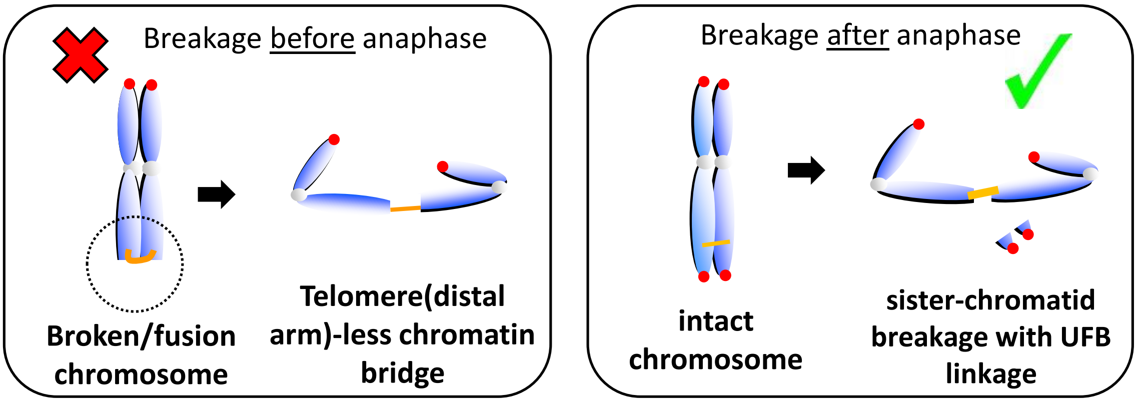

b

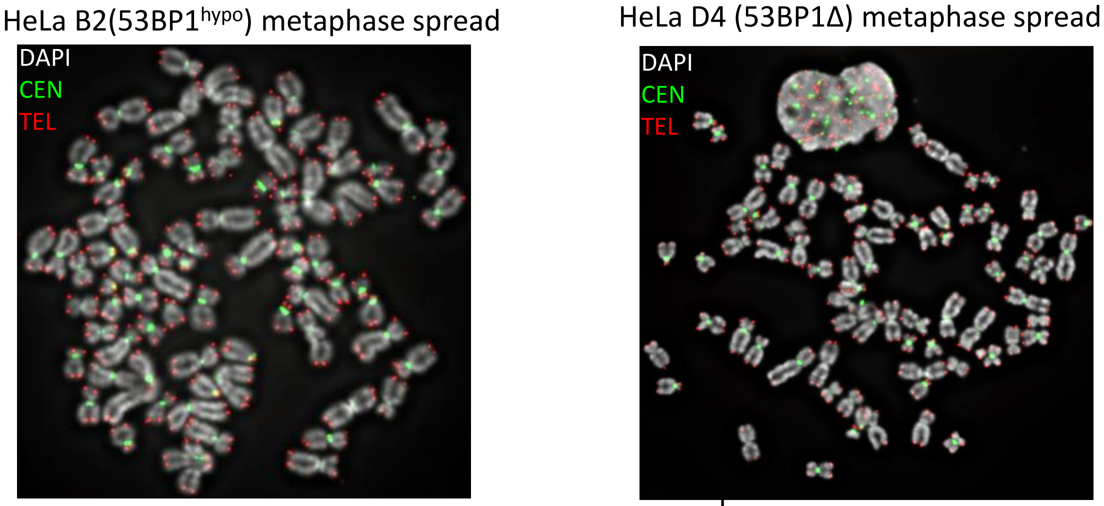

Sister-chromatid fusion

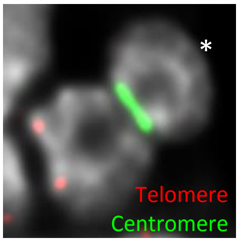

**HeLa B2(53BP1^{hypo})**	**Broken/fusion chromosome**
% metaphase spread	2.3±2% (2/84)
% chromosome	0.03±0.03% (2/6284)

**HeLa D4 (53BP1Δ)**	**Broken/fusion chromosome**
% metaphase spread	2.44±2.14% (4/88)
% chromosome	0.06±0.02% (4/6539)

c

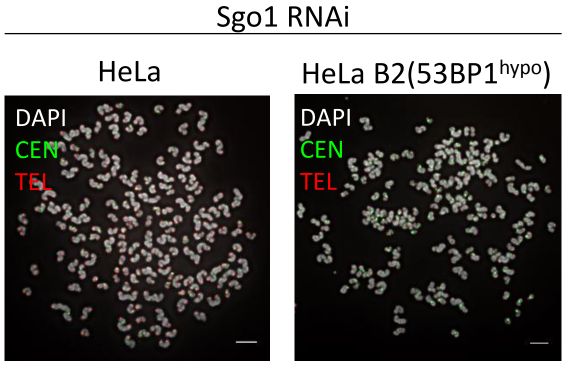

Non-intact chromatid

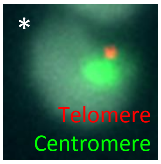

	**Non-intact chromatid**
**% metaphase spread**	HeLa: 10% (2/20)<br>B2: 15% (3/20)
**% chromosome**	HeLa: 0.06% (2/3262)<br>B2: 0.16% (5/3168)

d

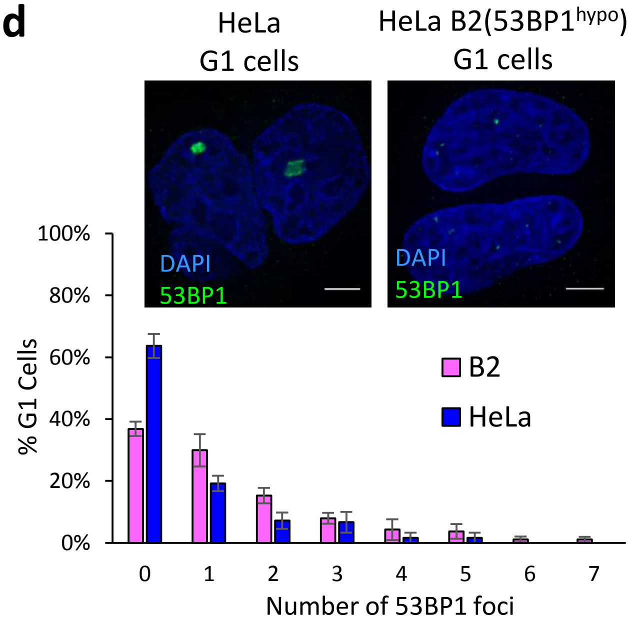

Supplementary Figure 9. 53BP1 depletion does not cause chromosomal breakage before anaphase, but lead to increased DNA damage in G1 population

(a) Two models depicting the generation of telomere/arm-less chromatin bridges in anaphase caused by sister-chromatid arm fusions (**Left**) or sister-chromatid rupture (**Right**).

(b) Quantitation of metaphase chromosomes with sister-chromatid arm fusion or arm breakage in HeLa B2 ($53BP1^{hypo}$) & D4 (53BP1Δ) mitotic cells. Representative FISH image showing metaphase chromosomes of HeLa B2 ($53BP1^{hypo}$) and D4 (53BP1Δ) labelling with centromere (green) and telomere (red) PNA probes. Note: all chromosomes contain a single centromere and two flanking sister telomeres. Inset demonstrating a rare example of a chromosome with sister-arm fusion: missing one telomere end (asterisk). A table illustrating the frequencies of metaphase spreads and chromosomes with sister-arm fusion events in HeLa B2 ($53BP1^{hypo}$) & D4 (53BP1Δ) mitotic cells. Over 6000 chromosomes from 88 metaphase spreads were counted in 3 independent experiments.

(c) Quantitation of single sister chromatid showing chromatid breakages in HeLa and $53BP1^{hypo}$ cells after knockdown of Sgo1 to induce premature sister chromatid separation. Over 3000 chromosomes from 20 metaphase spreads. Inset demonstrating an example of a non-intact chromatid with missing one telomere end (asterisk).

(d) 53BP1 nuclear body or foci were scored in HeLa and 53BP1hypo G1 cells. EdU pulse-labelling and CENPF were used as markers to identified S and G2 phase cells. 53BP1 foci were scored in G1 cells (absence of both above signals). Over 200 cells were counted in 3 independent experiments. Error bars represent s.d.

Supplementary Figure 10

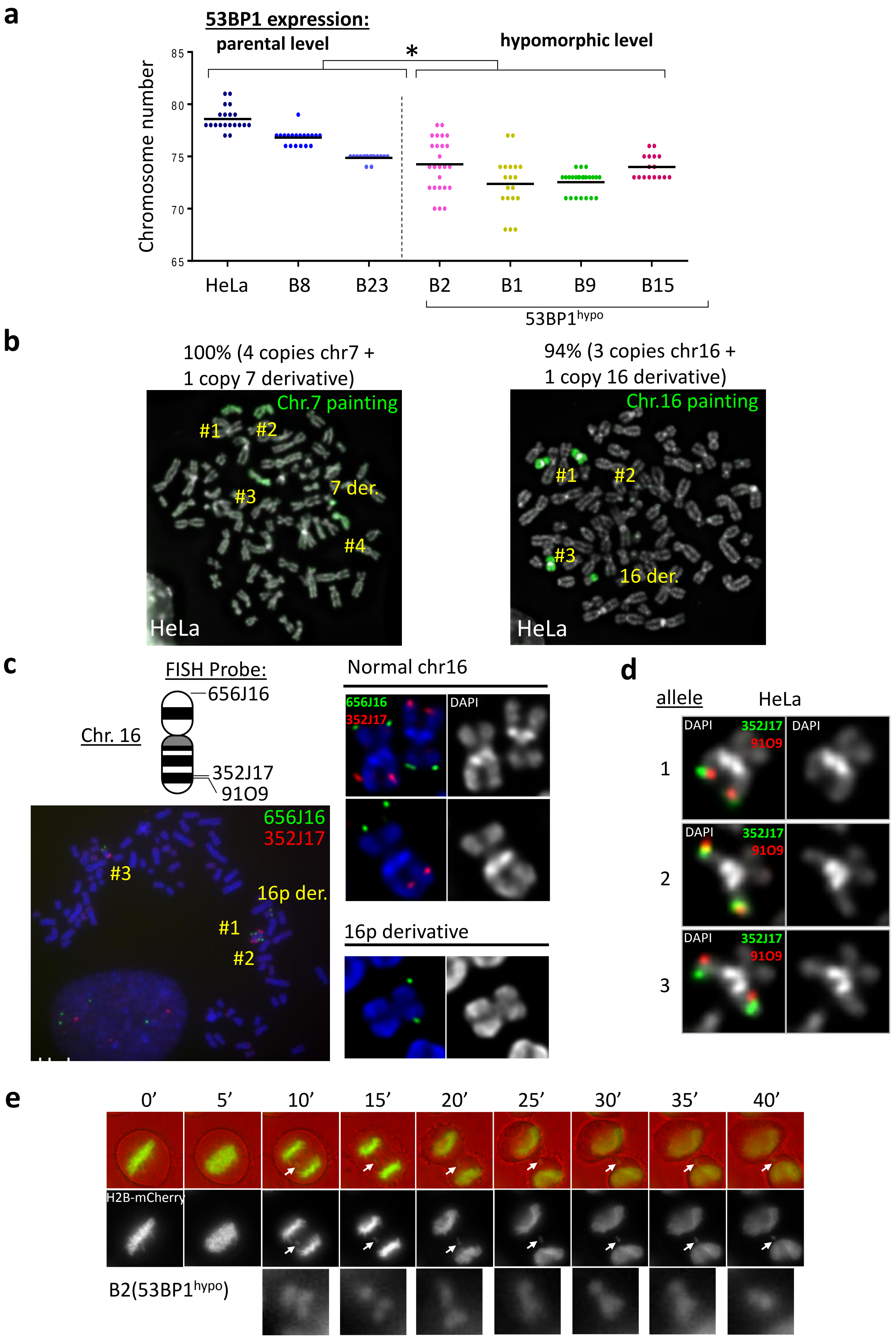

Supplementary Figure 10. HR-mediated sister chromatid bridging leads to numerical chromosomal rearrangements in 53BP1-depleted cancer cells.

(a) Numerical chromosome instability after 53BP1-depletion in HeLa cells. Note a decrease in the number of chromosomes in hypomorphic 53BP1 clones. 22 metaphase spreads from each cell line were scored. Statistical significance was determined by T-test (*, $p < 0.05$).

(b) Whole chromosome painting on chromosomes 7 and 16 was performed on metaphase spreads of HeLa cells. Four intact chromosome 7 plus a 7 derivative (7 der.) (100%) and three (94%), or two (6%) intact chromosome 16, plus a 16p derivative (16p der.) were found throughout the HeLa populations.

(c) Ideogram of chromosome 16 marking the positions of FISH BAC clones used. Dual-colour fluorescence in situ hybridization (FISH) was performed on metaphase spreads of HeLa cells. Three, or two intact chromosome 16, plus a 16p derivative (16p der.) were found throughout the HeLa population. **Right**: enlarged views of three intact copies of chr.16 and the 16p derivative in HeLa cells.

(d) HeLa metaphase chromosomes 16 display a normal FISH hybridisation pattern using two probes against FRA16D CFS (compared to Fig. 7b-c).

(e) Live-cell imaging of a HeLa B2 ($53BP1^{hypo}$) cell stably expressing H2B-mCherry progressing through mitosis. A lagging-chromatin pair (arrow) fails to disjoin and co-segregate to the same daughter offspring cell. Insets showed the enlarged images of the chromatin pair.

Supplementary Figure 11

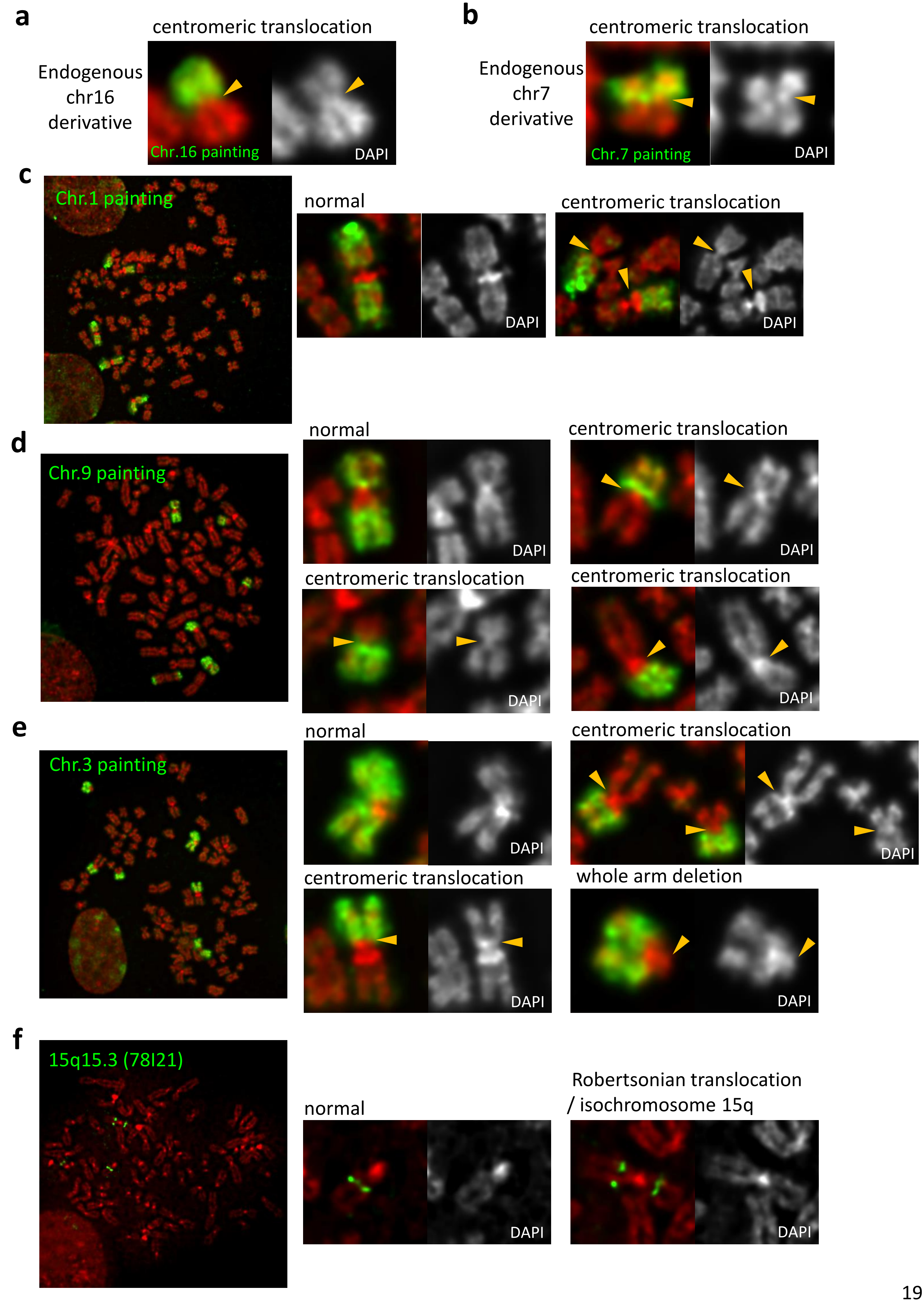

Supplementary Figure 11. HeLa genome harbours marker chromosomes of Robertsonian (-like) translocations.

(a) Whole chromosome 16 painting (green) shows centromere translocation involving chr16.
(b) Whole chromosome 7 painting (green) shows centromere translocation involving chr7.
(c) Whole chromosome 1 painting (green) reveals two marker chromosomes with centromere translocations involving chr1.
(d) Whole chromosome 9 painting (green) reveals three marker chromosomes with centromere translocations involving chr9.
(e) Whole chromosome 3 painting (green) reveals three marker chromosomes with centromere translocations involving chr9 and one with whole-arm deletion.
(f) Fluorescence in situ hybridization (FISH) using a 15q15.3 BAC clone probe (green) identified an isochromosome 15q or Robertsonian translocation.

Supplementary Figure 12

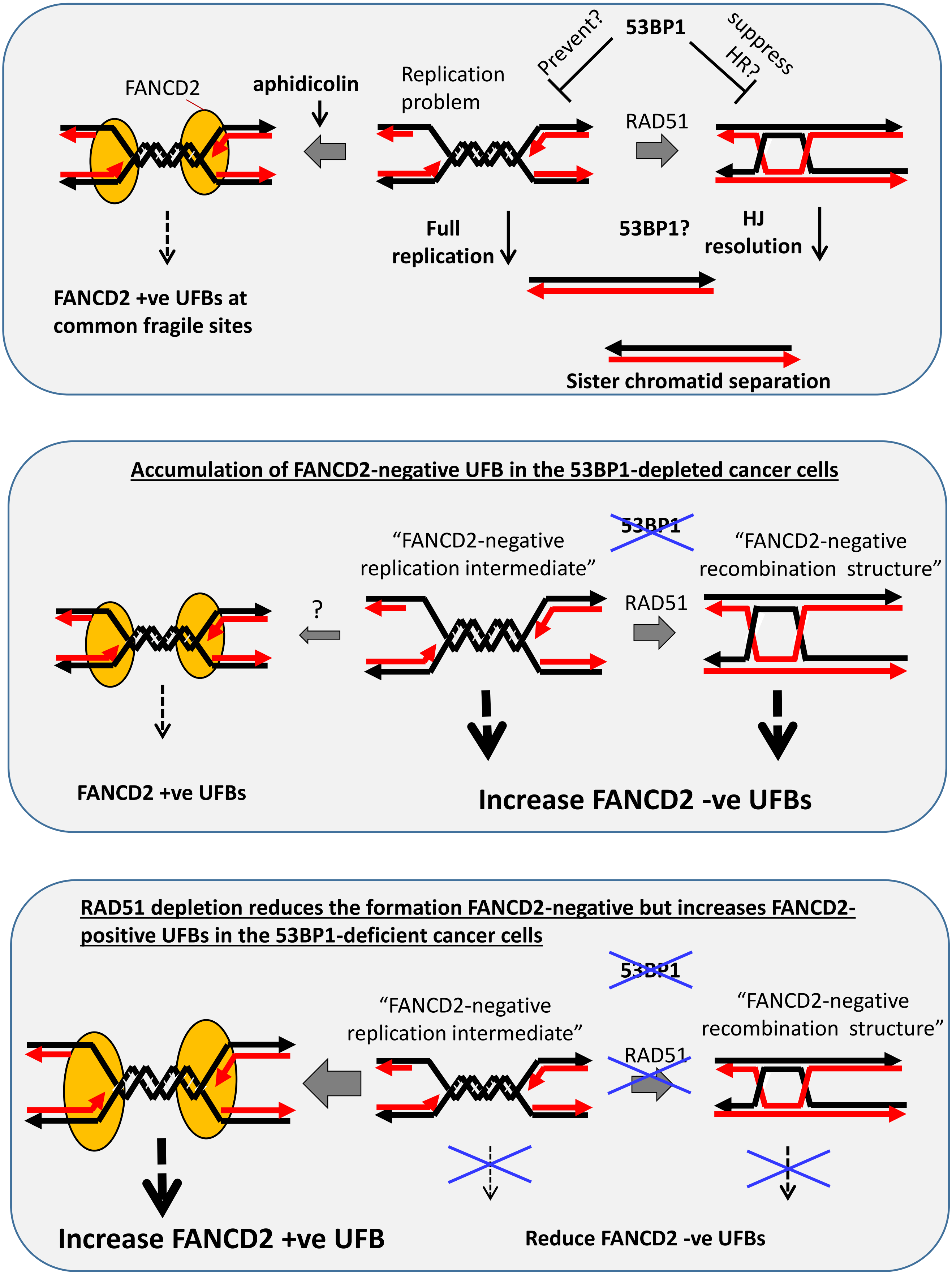

Supplementary Figure 12. A model depicts the possible mechanisms of the formation of sister chromatid intertwinements in 53BP1-depleted cancer cells.
